# A Comparison of Three Automated Nucleic Acid Extraction Systems for Human Stool Samples

**DOI:** 10.3390/microorganisms12122417

**Published:** 2024-11-25

**Authors:** Wit Thun Kwa, Choon Kiat Sim, Adrian Low, Jonathan Wei Jie Lee

**Affiliations:** 1 Centre for Translational Medicine, Department of Medicine, Yong Loo Lin School of Medicine, National University of Singapore, 14 Medical Drive, Singapore 117599, Singapore; kwa.w.t@nus.edu.sg (W.T.K.); cksim@nus.edu.sg (C.K.S.); adlow@nus.edu.sg (A.L.); 2 Institute for Health Innovation and Technology (iHealthtech), National University of Singapore, E7, 15 Kent Ridge Crescent, Singapore 119276, Singapore; 3 Division of Gastroenterology & Hepatology, Department of Medicine, National University Hospital, Singapore 119074, Singapore

**Keywords:** automated DNA extractor, ZymoBIOMICS mock community, bead-beating, fecal microbiota, KingFisher Apex, Maxwell RSC, GenePure Pro, human fecal samples

## Abstract

Automated nucleic acid extractors are useful instruments for the high-throughput processing of bio-samples and are expected to improve research throughput in addition to decreased inter-sample variability inherent to manual processing. We evaluated three commercial nucleic acid extractors Bioer GenePure Pro (Bioer Technology, Hangzhou, China), Maxwell RSC 16 (Promega Corporation, Madison, WI, USA), and KingFisher Apex (ThermoFisher Scientific, Waltham, MA, USA) based on their DNA yield, DNA purity, and 16S rRNA gene amplicon results using both human fecal samples and a mock community (ZymoBIOMICS Microbial Community Standard (Zymo Research Corp., Irvine, CA, USA)). Bead-beating provided incremental yield to effectively lyse and extract DNA from stool samples compared to lysis buffer alone. Differential abundance analysis and comparison of prevalent bacterial species revealed a greater representation of Gram-positive bacteria in samples subjected to mechanical lysis, regardless of sample type. All three commercial extractors had differences in terms of yield, inter-sample variability, and subsequent sequencing readouts, which we subsequently share in the paper and believe are significant considerations for all researchers undertaking human fecal microbiota research.

## 1. Introduction

Laboratory automation serves to streamline workflow and mitigate risk to the operator [1,2]. Manual sample processing is susceptible to human error and sample-sample variation, affecting the reproducibility of research [2,3]. Studies have also reported fewer contaminated samples from automated processes (e.g., PCR preparation and nucleic acid extraction) compared to manually processed tasks [4,5]. Apart from maintaining sample integrity, the automated instruments, when optimized, could process samples in a high throughput manner thus limiting the number of processing batches. Such batch effects could confound omics results making it difficult to differentiate real biological changes from biases despite efforts to reduce and minimize biases through sample randomization, and mathematical and statistical models [6,7,8,9]. Hence, laboratory automation could increase productivity and improve results quality among other benefits [2].

The quality of microbiome data depends on the reproducibility and robustness of the nucleic acid extraction method, but differences between DNA extraction kits can result in high variability between replicates of the same sample [10]. Automated nucleic acid extractors are marketed to reduce manual handling and, since their introduction in the early 2000s, have been implemented in forensic science and clinical studies for viral and bacterial pathogens [5,11,12,13]. Benchtop automated extractors are mainly liquid handlers that aliquot reagents and samples, perform nucleic acid using DNA binding magnetic beads, and have limited heating capacity to optimize enzymatic activity such as proteinase K. Most extractors are not equipped to perform bead-beating, which may compromise microbiome results as mechanical lysis is regarded as the gold standard to effectively lyse different microbial cell types, including spores, and is included in the International Human Microbiome Standards project standard operating procedures for fecal samples [14,15,16,17,18,19,20]. Therefore, a comparison between the automated nucleic acid extractors with and without bead-beating is warranted to ensure sufficient recovery of fecal microbiota in the DNA.

To our knowledge, few studies have compared the performance of automated nucleic acid extractors on human fecal samples [19,21]. In this study, we assessed the efficiency of three automated DNA extractors, namely, GenePure Pro (Bioer Technology, Hangzhou, China), Maxwell RSC 16 (Promega Corporation, Madison, WI, USA), and KingFisher Apex (ThermoFisher Scientific, Waltham, MA, USA), compared to a conventional column-based DNA extraction kit (MP-Biomedicals, Santa Ana, CA, USA). Furthermore, we compared the DNA yield, quality, and variation in alpha- and beta-diversity between extractors with and without bead-beating treatment.

## 2. Materials and Methods

### 2.1. Sample Collection and Processing

**Figure 1:**
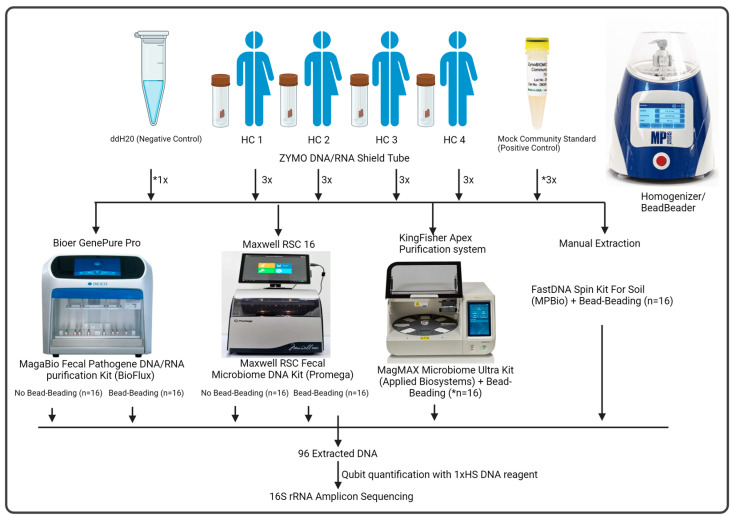
Experimental Design. Three homogenized stool and DNA shield mixtures were taken from each of the four healthy individuals (HC) as biological triplicates. The mock community is of eight bacterial species in equal DNA proportions (ZymoBIOMICS Microbial Community Standard), triplicates for all systems except the KingFisher system which used duplicate samples *. Molecular grade water was used as the negative control for all systems. The MP-Biomedicals (MP-Bio) FastPrep homogenizer was used for all samples that underwent bead-beating. The figure was created using BioRender.com (accessed on 18 November 2023).

This study used fecal samples from four healthy volunteers as biological samples and triplicate fecal aliquots as technical replicates for each volunteer per method (Figure 1). The fecal samples from each donor were collected in stool buckets and transported to the laboratory within 2 h. Stool samples were processed immediately in an anaerobic chamber (Coy; 91% N_2_, 5% CO_2_, 4% H_2_) upon delivery. One gram (*w*/*w*) of stool was aliquoted into a DNA/RNA Shield Fecal Collection Tube (Zymo Research Corp., Irvine, CA, USA) containing 9 mL of preservation reagent and vortexed then stored in a −80 °C freezer until DNA extraction (estimated preparation time ~20 min). The mock community used in this study is the ZymoBIOMICS Microbial Community Standard (Zymo Research Corp., Irvine, CA, USA) and the negative control is molecular-grade water (Cytiva, South Logan, UT, USA) (Figure 1). The fecal samples and mock community were processed as a batch per DNA extraction method. Table 1 details each DNA extraction method’s preparation and processing time.

### 2.2. DNA Extraction and Quantification

All samples were processed and extracted using different automated DNA extraction systems alongside manual extraction. The characteristics of automatic extractors and kits are described in Table 1. Before DNA extraction, frozen fecal samples were thawed at room temperature. The volumes taken for the fecal DNA shield mixture was 300 µL per replicate, the mock community was 75 µL, and the negative control was 300 µL of molecular water. The FastPrep-24 5G Bead Beating Grinder and lysis system (MP-Biomedicals, Santa Ana, CA, USA) was used for homogenization at 6.0 m/s for 40 s.

The DNA concentration and quality were measured using a Qubit 4 fluorometer with a dsDNA HS assay kit (Life Technologies Corporation, Carlsbad, CA, USA) and a NanoDrop One spectrophotometer (ThermoFisher Scientific, Waltham, MA, USA), respectively. All extracted DNA was stored at −80 °C before library preparation for 16S rRNA amplicon sequencing. Detailed extraction protocols for automated extractors are described in Appendix A without deviation unless stated otherwise. All centrifugations were performed at 14,000× *g* unless stated otherwise.

#### FastDNA Spin Kit for Soil (MP-Biomedical Catalog No: 116560200)

Samples were added to lysing matrix E tubes each containing 978 µL of Sodium Phosphate Buffer and 122 µL of MT Buffer (MP-Biomedicals). The lysate was centrifuged for 15 min. The supernatant was transferred to a 2 mL microtube with the addition of 250 µL of protein participated solution (PPS) mixed and incubated on ice for 5 min. Thereafter, the mixture was centrifuged for 5 min and transferred to a 5 mL tube followed by the addition of 1 mL binding matrix solution, inverted twice, and left to stand for 3 min. The top 500 µL of supernatant was removed and the remaining binding matrix was gently mixed and 600 µL transferred to a SPIN Filter tube. The SPIN filter tube was centrifuged for 1 min, washed with 500 µL SEWS-M buffer, centrifuged for 1 min, and dried by centrifugation for another 2 min. DNA was incubated with 70 µL of pre-heated (55 °C for 5 min) DES elution buffer and centrifuged for 2 min to collect eluted DNA.

### 2.3. 16S Amplicon Sequencing Library Preparation and Analysis

All purified DNA (*n* = 96) were sent and underwent library preparation (Nextera DNA Library Prep Kit; Illumina, San Diego, CA, USA) and 16S amplicon sequencing (Illumina MiSeq 2 × 300 bp paired-end sequencing). Primers 338F-5’ CCTACGGRRBGCASCAGKVRVGAAT and 806R-5’GGACTACNVGGGTWTCTAATCC were used to amplify the V3–V4 region of the 16S rRNA gene with initial denaturation at 95 °C for 3 min, followed by 28 cycles 95 °C for 30 s, 55 °C for 30 s, and 72 °C for 30 s, with final elongation at 72 °C for 5 min.

All the sequenced samples, including kit controls, were analyzed to evaluate the effect of the different nucleic extraction systems on fecal microbiota. For the mock community, a total of 1,700,732 denoised, merged, and non-chimera reads were obtained from 3,232,230 raw paired-end reads. The number of denoised reads ranged from 89,225 to 164,487. For the fecal microbiota, a total of 9,272,449 denoised, merged, and non-chimera reads were obtained from 15,160,148 raw paired-end joined reads. The number of denoised reads ranged from 101,108 to 188,910.

For each software, default parameters were used unless stated otherwise. Raw fastq reads were processed by cutadapt (version 4.2) to remove primer sequences [22]. The trimmed reads then underwent DADA2 (version 1.28.0) processing to infer amplicon sequence variants (ASVs) and taxonomic classification against the SILVA SSU release 138 non-redundant 99% database [23,24]. Shannon diversity and richness were calculated using “estimate_richness” from the phyloseq R package (version 1.48.0) [25]. Aitchison distance was calculated by first performing a centered log ratio (CLR) transformation, followed by “distance” from the phyloseq package. Permutational multivariate analysis of variance (PERMANOVA) and pairwise PERMANOVA were performed using the adonis2 function (vegan package version 2.6-8) and pairwise adonis function (pairwiseAdonis package version 0.3), respectively [26,27]. Differential taxa abundance was performed using MaAsLin2 with no random effects and extraction method as a group as the fixed effect with manual extraction set as the reference group and the default Wald test with Benjamini-Hochberg *p*-value correction as statistical test [28]. Prevalent genera are based on the sum of detects (absence-presence) for each species and filtered to identify genera that showed a difference in detection for ≥4 samples (≥33.33%) between the lowest detect and highest detect across the extraction methods. The heatmap shows all the prevalent genera identified and was generated using the pheatmap function (version 1.0.12) that ran in R (version 4.3.3). Statistics were performed using the Kruskal-Wallis test followed by post-hoc Dunn’s test with Benjamini-Hochberg correction using R (version 4.3.3). Pairwise statistical tests of alpha-diversity indices, beta-diversity matrix, and the relative abundance of bacterial species between each were performed using Wilcoxon signed-rank tests with Bonferroni correction for multiple testing. Gram-stain phenotype for each taxon is attained from the BacDive online database based on the type strain for species-level assignment or a consensus of three type species of human gastrointestinal tract sourced, when available for the genus level taxon [29].

## 3. Results

### 3.1. Comparison of DNA Yield and Purity of Fecal and Mock Microbiotas Between the DNA Extraction Methods

**Figure 2:**
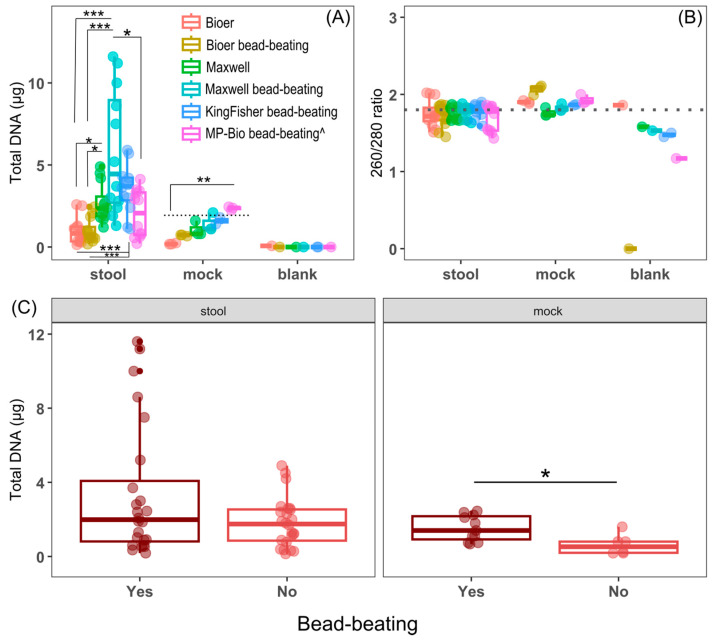
Quantity and purity of DNA extracted using different nucleic acid extraction systems. (**A**) Total DNA amount (µg) of samples grouped by DNA extraction method. MP-Biomedicals is abbreviated MP-Bio. (^) denotes manual extraction method. The theoretical amount of DNA (2 µg) in the mock sample is shown by the dotted line. (**B**) DNA purity is based on 260/280 absorbance ratio. The ideal DNA to protein ratio of 1.8 is shown as a dotted line. Blank indicates molecular water samples as negative control. (**C**) Total DNA extracted from different sample types with and without bead-beating. “Yes” or “No” consists of 24 fecal samples for Bioer and Maxwell and 6 mock samples for Bioer and Maxwell. Boxplots show the median, first, and third quartiles where whiskers extend from the hinge no further than 1.5× the interquartile range. The number of stool samples for each extraction method (*n* = 12) consists of biological replicates (*n* = 4) and technical triplicates (*n* = 3). The mock community consists of biological triplicates for each extraction system except for KingFisher where samples were biological duplicates (*n* = 2) owing to insufficient volume. The kit control consists of a single sample for each of the methods except KingFisher (*n* = 2). The post-hoc Dunn’s test with Benjamini-Hochberg *p*-value correction was performed for stool and mock samples where (*) denotes *p*-adjusted ≤ 0.05, (**) denotes *p*-adjusted ≤ 0.01, and (***) denotes *p*-adjusted ≤ 0.001. Overlapping horizontal black lines share the same statistical significance.

We compared the quantity and purity of DNA extracted using three automated DNA extraction systems with and without bead-beating except for KingFisher (bead-beating only) and a conventional column-based extraction kit (MP-Biomedicals) (Figure 1). Figure 1 shows the sample composition (*n* = 16) per extraction run consisting of triplicate samples from each of the four subjects (*n* = 12), mock community (*n* = 3), and one negative control (*n* = 1). It should be noted that there was only sufficient mock culture for duplicate samples (*n* = 2) for the KingFisher system with an additional negative control (*n* = 2) included to make up the 16 samples. Two different sample types were tested, stool samples from four individuals and a low-diversity mock community comprising eight bacterial species (three Gram-negative and five Gram-positive) and two yeasts (ZymoBIOMICS Microbial Community Standard; Zymo Research Corp., Irvine, CA, USA) to test for extraction biases (Figure 1). DNA quantified using a fluorescent dye specific to dsDNA showed differential yields were obtained from fecal samples among the systems (chi-squared = 14.6601, df = 5, *p*-value = 0.01, Kruskal Wallis) (Figure 2A). To circumvent the differences in the elution buffer, we compared the total DNA extracted from each system.

Regarding fecal samples, the Bioer system regardless of bead-beating yielded lower total DNA compared to most systems (Figure 2A). The Maxwell and KingFisher systems shared similar yields, but only the former with bead-beating showed a statistically significant higher yield than the conventional kit (Figure 2A). However, it should be noted that the variation in yield is also the highest for Maxwell with bead-beating. In contrast, the Bioer system, regardless of bead-beating, had the lowest variation in yield (Figure 2A).

For the mock culture, Bioer without bead-beating yielded the lowest amount of DNA, 0.198 ± 0.04 (SD) µg, which is significantly less than the conventional kit (Figure 2A). The remaining systems yielded similar median DNA that is not too different from the expected yield of 2 µg (Zymo) (Figure 2A). Similar to the fecal samples, Maxwell with bead-beating exhibited the highest variability in DNA yield for the mock community. No appreciable DNA was measured in the kit controls of sterile water except for a single Bioer, which extracted a low amount of DNA (70 ng). Based on the ratio of absorbance of DNA (260 nm) and protein (280 nm) as an indication of DNA purity, there was no statistically significant difference in the ratios between the methods for the stool or mock samples (Figure 2B). When we compared the DNA yield between samples subjected to bead-beating using the Bioer and Maxwell systems to samples without bead-beating, there was a significantly greater DNA yield in mechanical lysed mock samples than mock samples without bead-beating (*p*-adjusted = 0.0172, Dunn’s test with Benjamini-Hochberg adjustment, Figure 2C). However, a similar difference was not observed in the more heterogeneous fecal samples (Figure 2C).

### 3.2. Differences in the Mock Community of Nucleic Acid Extractors

**Figure 3:**
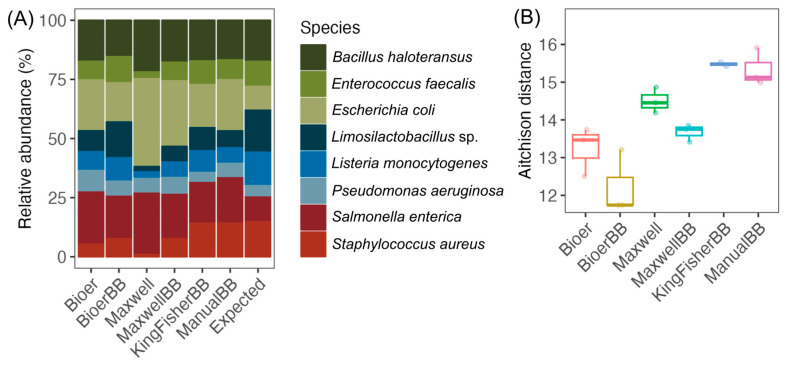
Mock community analysis of samples extracted using different DNA extraction systems. (**A**) A stacked barplot of the mock ZymoBIOMICS Microbial Community Standard that consists of eight bacterial species grouped by DNA extraction system. The expected microbiota is based on the theoretical composition (16S rRNA gene copies) of each bacterial species as stated in the manufacturer’s protocol. (**B**) Aitchison distance of mock microbiota relative to the expected mock microbiota. The mock community consists of triplicate samples for each extraction method, except for KingFisher, with duplicate samples.

The relative abundances of the eight bacterial species in the “expected” sample are based on the theoretical number of 16S rRNA genes as provided by the manufacturer (Figure 3A). All eight bacterial species of the mock community were extracted in each extraction method/system, but the difference in proportion to the “expected” mock community suggests there may be different extraction efficiencies for the varying cell types (Figure 3A). The Bioer and Maxwell kits with bead-beating improved the recovery for *Enterococcus faecalis* (Gram-positive), *Limosilactobacillus fermentum* (Gram-negative), and *Staphylococcus aureus* (Gram-positive) compared to the respective kits without bead-beating (Figure 3A). A notable difference in the ratio of *Escherichia coli* (Gram-negative) to *Salmonella enterica* (Gram-negative) (1:1; expected) can be observed in both Maxwell methods, where *E. coli* is disproportionally greater in relative abundance (>10%) than *S. enterica*, which suggests a kit effect on DNA extraction. The microbiota extracted using the KingFisher and conventional kit showed similar community structure but with a lower proportion of *Limosilactobacillus fermentum* (Gram-negative) and *Listeria monocytogenes* (Gram-positive) compared to the expected microbiota (Figure 3A). The principal component analysis (PCA) plot showed that differing methods using the same instrument clustered closer together compared to other instruments or manual extraction (Figure S1). The Bioer system, with and without bead-beating, showed similar variation in Aitchinson distance among the paired mock samples (*n* = 3) compared to the manual bead-beating kit (MP-Biomedicals) (Figure 3B). While there was no statistically significant difference (*p*-adjusted > 0.05; pairwise PERMANOVA) in the Aitchison distance between any pair of systems, the system with the closest Aitchison distance to the conventional kit was KingFisher followed by Maxwell and Bioer, regardless of cell lysis method (Figure 3B and Figure S1).

### 3.3. Alpha-Diversity of Fecal Microbiota Between Nucleic Acid Extractors

**Figure 4:**
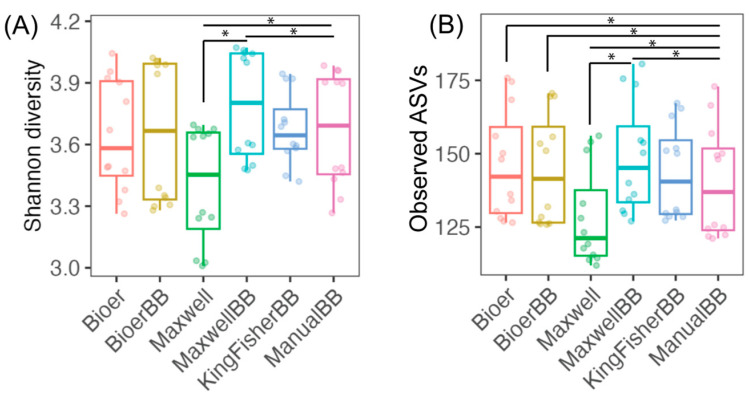
The alpha-diversity measure of fecal microbiota grouped by DNA extraction system. (**A**) Shannon diversity index and (**B**) Observed ASV richness of the six DNA extraction systems. Boxplots show the median, first, and third quartiles where whiskers extend from the hinge no further than 1.5× the interquartile range. The number of stool samples for each extraction method (*n* = 12) consists of biological replicates (*n* = 4) and technical triplicates (*n* = 3). Wilcoxon signed-rank test was performed where (*) denotes *p*-adjusted ≤ 0.05.

Figure 4 shows the Shannon diversity index and species richness of fecal microbiota grouped by extraction method. Maxwell without bead-beating had the lowest median Shannon diversity and species richness (Figure 4). In contrast, Maxwell with bead-beating had the highest median Shannon diversity and species richness (Figure 4B). Maxwell with bead-beating showed a similar Shannon diversity index and species richness to the Bioer system with and without bead-beating. The KingFisher and conventional kit extracted DNA showed comparable Shannon diversity and species richness (Figure 4B).

### 3.4. Variability of Microbiota Between Nucleic Acid Extraction Systems

**Figure 5:**
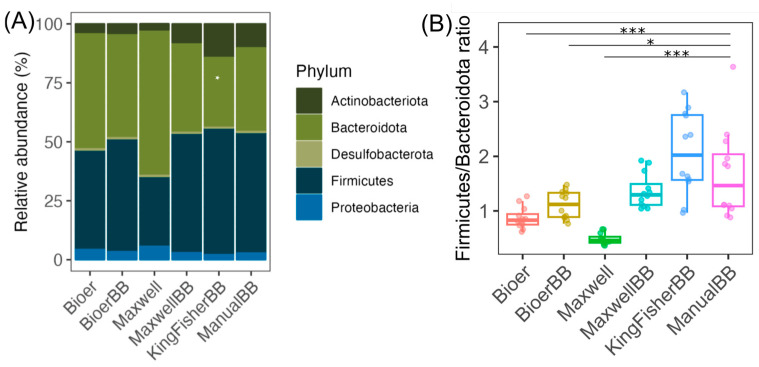
Fecal microbiota analysis grouped by DNA extraction system. (**A**) A stacked barplot of the fecal microbiota at the phylum level. (**B**) The ratio of Firmicutes to Bacteroidota grouped by DNA extraction system. Boxplots show the median, first, and third quartiles where whiskers extend from the hinge no further than 1.5× the interquartile range. The number of stool samples for each extraction method (*n* = 12) consists of biological replicates (*n* = 4) and technical triplicates (*n* = 3). The (*) denotes *p*-adjusted ≤ 0.05, and (***) denotes *p*-adjusted ≤ 0.001, based on the Wald test with Benjamini-Hochberg *p*-value adjustment implemented in MaAsLin 2.

**Figure 6:**
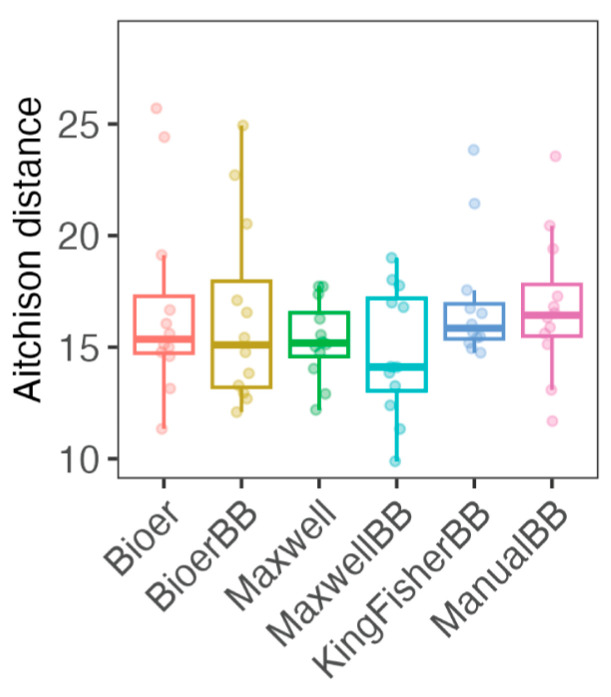
Reproducibility of extraction method. Each boxplot represents Aitchison distances among the biological replicates within each extraction method.

The human fecal microbiota is dominated by ASVs of Firmicutes (mostly Gram-positive) and Bacteroidota (mostly Gram-negative) with Actinobacteriota (Gram-positive), Desulfobacterota (Gram-negative) and Proteobacteria (mostly Gram-negative) as the minor phyla (Figure 5A). Comparison of fecal microbiota at the phylum level showed disproportional community structure for Bioer and Maxwell without bead-beating, which have a high proportion of Bacteroidota in the samples (Figure 5A). In contrast, Firmicutes was the major phylum in Bioer, Maxwell, KingFisher, or manual extraction in the microbiota (Figure 5A). This is evident in the Firmicutes (mostly Gram-positive bacteria) to Bacteroidota (mostly Gram-negative bacteria) ratio with statistically significantly higher ratios in the majority of the bead-beaten samples, compared to the non-bead-beaten samples (Figure 5B). Among the samples subjected to bead-beating, KingFisher had the highest median ratio and largest variation, although not significantly different from the samples extracted using the other systems (Figure 5B). Based on the Aitchison distance, there was no statistically significant difference in Aitchison distance among the methods, owing to strong inter-biological sample variation, suggesting that the majority of ASVs are recovered regardless of systems and methods (Figure 6 and Figure S2 for PCA plot). When we examined the technical replicates grouped by individual (HC1, HC2, HC3, and HC4), Maxwell without bead-beating samples clustered furthest from the other methods, albeit not to a statistically significant degree (pairwise-PERMANOVA), owing to low statistical power due to small sample size (*n* = 3) (Figure S2).

### 3.5. A Higher Relative Abundance of Gram-Positive Bacteria in Bead-Beaten Fecal Samples

**Figure 7:**
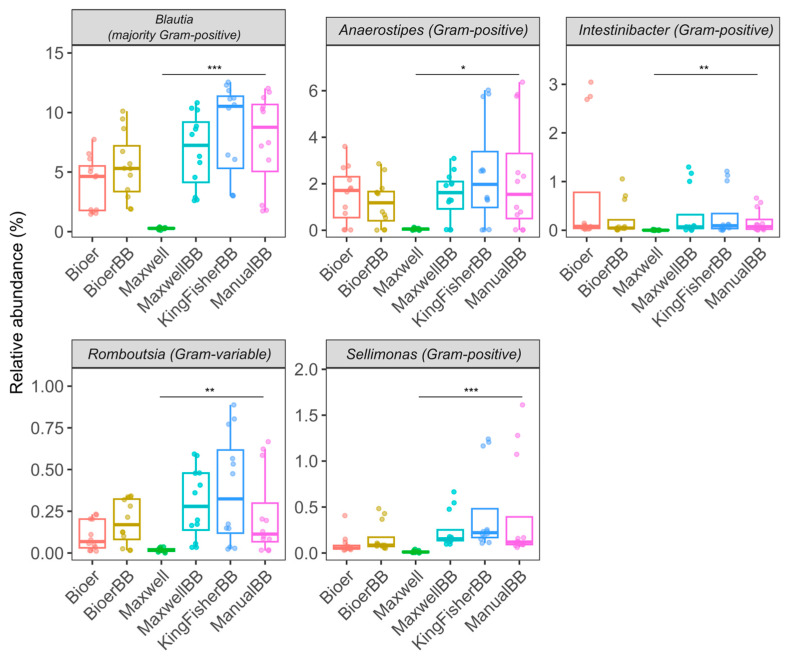
Five bacterial genera were differentially abundant between two or more DNA extraction systems analyzed using MaAsLin2. The number of stool samples for each extraction method (*n* = 12) consists of biological replicates (*n* = 4) and technical triplicates (*n* = 3). The (*) denotes *p*-adjusted ≤ 0.05, (**) denotes *p*-adjusted ≤ 0.01, and (***) denotes *p*-adjusted ≤ 0.001, based on the Wald test with Benjamini-Hochberg *p*-value adjustment implemented in MaAsLin2.

We identified five bacterial genera that were differentially abundant between two DNA extraction systems (Figure 7). The Maxwell kit had significantly lower relative abundances for all of the five genera compared to the manual bead-beating kit (Figure 7). The systems incorporating bead-beating generally showed better recovery for *Blautia* (majority Gram-positive), *Anaerostipes* (Gram-positive)*, Romboutsia* (Gram-variable)*, Sellimonas* (Gram-positive) and *Intestinibacter* (Gram-positive) ASVs (Figure 7).

### 3.6. Heterogeneity in Prevalent Bacterial Genera Among Extraction Methods

**Figure 8:**
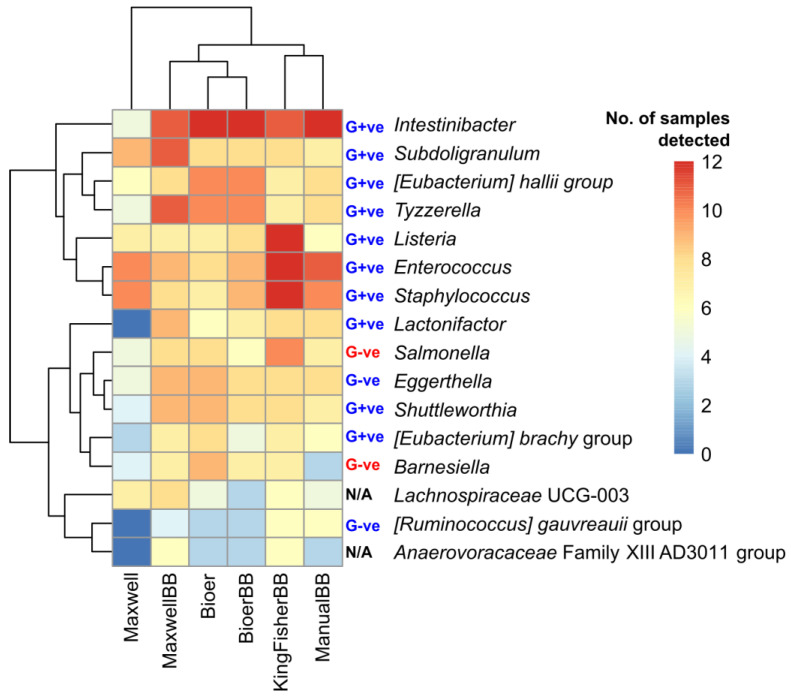
A heatmap showing the prevalent genera across the extraction methods. Bacteria were identified based on a minimum difference in detection (*n* = 4) between any two extraction methods. The number of stool samples for each extraction method (*n* = 12) consists of biological replicates (*n* = 4) and technical triplicates (*n* = 3). Hierarchical clustering is based on Euclidean distances. G+ve denotes Gram-positive, G−ve denotes Gram-negative and N/A denotes phenotype is not available. Gram stain phenotype is based on the type species of each genus or consensus of three species for family-level phylogeny.

We identified 16 prevalent bacterial genera that showed a high difference in detection (minimum four sample differences) between any two methods. From the heatmap, Maxwell without bead-beating had the highest number of low and non-detections (four or fewer samples) genera among the methods (Figure 8). The incorporation of bead-beating into the Maxwell system improved the detection of those bacterial genera with low prevalence and with two genera (*Tyzzera* and *Subdoligranulum*.) that showed improved prevalence across the 12 samples compared to any other method. Based on hierarchical clustering, the Bioer system with and without bead-beating clustered together indicating high similarity in prevalent genera profile with at least two bacterial genera of *[Ruminococcus] gauvreauii* and *Anaerovoracaceae* Family XIII AD3011 groups with low prevalence (2 samples each). KingFisher is clustered with manual extraction and had the most genera detected in six or more samples.

## 4. Discussion

We compared the performance of three automated nucleic acid extractors to a conventional column-based kit. We also studied using a high-diversity complex fecal and low-diversity mock community. Our study is consistent with studies that showed greater yield and recovery of Gram-positive bacteria using mechanical and chemical lysis methods compared to other lysis methods [30,31]. Improvement in bacteria recovery is most evident in the Maxwell system with and without bead beating, where there were higher relative abundances of differentially abundant bacteria and fewer low detections in the bead-beaten samples. A study similar to ours which compared different automated DNA extraction systems showed similar DNA yields among the methods tested, but significantly greater alpha diversity in mechanically lysed samples compared to kits without bead-beating [19]. While this was true for Maxwell with bead-beating and Maxwell without bead-beating, Bioer samples showed similar alpha-diversity and prevalent bacteria regardless of bead-beating, indicating that lysis buffer alone may suffice when using the Bioer kit. The effect of sub-optimal cell lysis can be seen in the Firmicutes to Bacteroidota ratio, where lower ratios were observed in the samples not subjected to mechanical lysis. Suboptimal cell lysis and DNA extraction could lead to incorrect interpretation of microbiome data where the Firmicutes to Bacteroidota ratio has been shown to increase in obesogenic hosts [32,33]. This is also congruent when we compared the prevalent bacterial genera, where the Maxwell kit without bead-beating showed poor recovery for several gut microbes. All the systems tested showed relatively pure DNA and were suitable for 16S rRNA gene amplicon sequencing, and most samples would be of sufficient quality for long-read sequencing 260/280 ratio between 1.8 and 2.0.

Automated extractors that use magnetic nucleic acid binding kits may differ in performance. A field deployable semi-automated nucleic acid extractor that uses paramagnetic beads for DNA purification has been compared to the Maxwell RSC 16 system and the former showed greater recovery of bacterial species from a low biomass (10^7^ cells) mock community than the latter system [34]. Our study has not tested such low biomass, as our intended use of the DNA extractor is for human fecal samples expected to be in the magnitude of 10^10^ cells per gram (wet weight) of stool [35].

Among the extractor systems, KingFisher exhibited the lowest variation in DNA yield, fewest low detection species, and variation in beta diversity for fecal and mock microbiotas of all the systems, including the conventional kit and, in this regard, maybe the most reliable. While the Maxwell system with bead-beating yields the highest median DNA and alpha diversity, it was also the most variable for the fecal and mock samples. Interestingly, the Firmicutes to Bacteroidota ratio of fecal microbiota extracted using the Maxwell system was lower in variation compared to the KingFisher extracted fecal microbiota, which indicates that ASVs of the two major phyla were more reproducible using the former system.

The high median alpha-diversity indices in the Maxwell system with bead-beating are due to the recovery of more ASVs of Actinobacteriota (Gram-positive) compared to most systems, especially Bioer. Although Actinobacteriota are not as predominant as bacterial species from Firmicutes or Bacteroidota, members such as *Bifidobacteria*, *Propionibacterium*, and *Corynebacterium* have been postulated to maintain gut homeostasis through the production of metabolites such as short-chain fatty acids and various oligosaccharides [36]. The low DNA yield obtained from the Bioer system, particularly in the fecal samples not bead-beaten, clearly had different microbiota compositional structures (low Firmicutes to Bacteroidota ratio) compared to the other systems.

## 5. Limitations

The result of this study is constrained by several factors. First, this study lacks a direct comparison between magnetic bead-based and silica column-based DNA extraction methods under identical buffer conditions. Although DNA yield and purity appeared comparable in our tests, differences in microbiota recovery could not be fully assessed due to potential variability in lysis efficiency and DNA binding between kits. Additionally, the study does not account for kit-specific challenges, such as column blockage, which may affect reproducibility and scalability in high-throughput settings [21,37]. Future research should standardize buffer conditions to better understand the impact of matrix type on DNA extraction efficiency and microbiota profiling.

Second, we have also not assessed the variability of manual processing by different operators that may bring about a larger variation in DNA yield and microbiota composition. The variation we reported could be attributed to variation in 16S amplicon sequencing, which is affected by PCR biases but we have limited the variability by keeping to the same PCR condition, library preparation kit, and same sequencing run [38,39]. Another limiting factor is the small sample size, particularly of the mock community, and the lack of replicates for the negative control (nuclease-free water). The number of samples we could extract at one time is limited by the instrument; different models that can handle larger sample sizes would need to be tested for variation. This would be important for laboratories looking to extract larger sample sizes and minimize batch effects [8].

Third, while we have examined the changes in microbiota among the automated extractors based on 16S amplicon sequencing results, shotgun metagenomics sequences would likely yield a similar outcome in terms of the underrepresentation of Gram-positive bacteria. One metagenomic study of two human cohorts with large sample sizes (*n* ≥ 292) extracted using a bead-based manual kit and an automated primarily heat-lysis extraction kit reported a similar under-representation of Gram-positive bacteria in the latter system [21]. As there are fundamental differences between metagenomics and 16S amplicon sequencing such as PCR and primer biases [40], with sufficient sequencing depth, metagenomics has been reported to provide better resolution compared to 16S amplicon sequencing [41]. Hence, it would be prudent to evaluate the metagenomic profile of DNA purified using automated systems.

## 6. Conclusions

In conclusion, this study demonstrated that despite similar DNA quality and quantity across of the methods, automated DNA extraction systems may display dissimilar microbiota composition and species recovery. These results should be taken into consideration by researchers undertaking human fecal microbiota research using automated DNA extractors.

## Figures and Tables

**Table 1:** The characteristics of the automatic DNA extractors and the kits used.

Characteristics	Genepure Pro	Maxwell RSC 16	King Fisher Apex	Manual Extraction
Manufacturer	Bioer	Promega	Applied Biosystem	N/A
Methods	Magnetic Bead-Based	Magnetic Bead-Based	Magnetic Bead-Based	Spin Columns
Format	96 Wells Pre-Packed Plate	Individual Pre-Packed Cartridge	96 wells Self-Filled Plate	Individual Self-Filled
Sample Volume	300 µL	300 µL	300 µL	300 µL
Throughput (samples/run)	1–32	1–16	1–96	Variable
Automation Level	Semi-automatic	Semi-automatic	Semi-automatic	Manual
Preparation Time (Based on 16 samples)	~25 min	~35 min	~40 min	N/A
Processing Time (Based on 16 samples)	~35 min	~42 min	~40 min	~100 min
Sterilization	Built-in UV	Built-in UV	Built-in UV	N/A
Type of Sample	Stool, Body Fluid	Stool, Body Fluid	Stool, Body Fluid	Stool, Soil
Kits Used	MagaBio Fecal Pathogens DNA Purification Kit (Bioer Technology, Hangzhou, China)	Maxwell RSC Fecal Microbiome DNA kit (Promega Corporation, Madison, WI, USA)	MagMAX Microbiome Ultra Kit (ThermoFisher Scientific, Waltham, MA, USA)	FastDNA Spin Kit for Soil (MP-Biomedicals, Santa Ana, CA, USA)
Homogenizer (Bead-Beating) Needed	Yes/No	Yes/No	Yes	Yes
Estimated Elution volume	50 µL	50–100 µL	50–200 µL	50–100 µL
Total Raw Reads for Stool Samples				
Without Bead-Beating	1,807,207	1,730,795	N/A	N/A
With Bead-Beating	1,482,643	1,753,841	1,223,111	1,274,852

Abbreviation: N/A = Not Applicable.

## Data Availability

Raw Illumina fastq files have been deposited in NCBI and assigned the BioProject number PRJNA1084203 and Biosample accession numbers SAMN40274884 to SAMN40274979.

## Supplementary Materials

The following supporting information can be downloaded at: https://www.mdpi.com/article/10.3390/microorganisms12122417/s1, Figure S1: Principal component analysis (PCA) plot based on Aitchison distance of the mock community using different methods; Figure S2: Principal component analysis (PCA) plots based on Aitchison distance among the fecal samples of different methods.

## Appendix A

Automatic DNA extractors, protocols, and relative kits.

### Appendix A.1. Bioer GenePure Pro Nucleic Acid Purification System (Catalog No. NPA-32P)

The MagaBio fecal pathogens DNA/RNA purification kit (Bioer) was used with the Bioer GenePure Pro nucleic acid automation purification system. Each MegaBio kit is designed for 16 samples on a 96-well plate prefilled with buffers in designated wells, using an 8-channel pipette. Samples were transferred to tubes with grinding beads, 200 µL of lysis buffer, and 10 µL Proteinase K. Samples subjected to bead-beating were homogenized followed by incubation at 65 °C for 10 min. After incubation, 70 µL DA buffer was added to each sample, vortexed for 30 s, and placed on ice for 5 min. Samples were centrifuged at 12,000× *g* for 5 min and 350 µL of supernatant were transferred to the sample well of a 96 well-plate for automated DNA purification. The manufacturer’s preset program had the following procedures, 20 s of magnetic beads mixing, 10 min of binding, and three washes with incubation periods of 3 min for the first wash and two minutes for the remaining washes followed by 5 min of incubation at 65 °C with 50 µL elution buffer but estimated 30–40 µL was available for transfer into a new 1.5 mL microtube. The total machine time was approximately 35 min.

### Appendix A.2. Promega Maxwell RSC DNA/RNA Extraction System (Catalog No: AS4500)

The Maxwell RSC Faecal Microbiome DNA kit (Promega, Catalog no: AS1700) was used with the Maxwell RSC 16 DNA/RNA extraction system (16 samples per run). Each kit contained 48 Maxwell RSC cartridges for 48 samples. All samples received 1 mL lysis buffer and 40 µL Proteinase K, proceeded by bead-beating for selected samples. Thereafter, all samples were placed at 95 °C for 5 min, then, cooled down at room temperature for 2 min, and vortexed for 1 min before incubation at 56 °C for 5 min. Samples were centrifuged for 5 min at room temperature before 300 µL per lysate was transferred to a Maxwell RSC cartridge for automated DNA purification.

The manufacturer’s preset program is described briefly, lysates were mixed with magnetic beads for 20 s, allowed to bind for 10 min, and washed thrice with wash buffer with incubation periods of 3 min for the first wash and two minutes for the remaining washes followed by 5 min incubation at 65 °C with 100 µL elution buffer. The total machine time was approximately 35 min. The plate was removed from the machine, the lid covered and samples with visible paramagnetic beads carried over were put on a magnetic rack, allowed to stand for 5 min and DNA dissolved in elution buffer was transferred into a new 1.5 mL microtube.

### Appendix A.3. KingFisher Apex Purification Systems (ThermoFisher Scientific), (Catalog No: 5400910)

The KingFisher Apex system referred to as KingFisher hereon is designed for 24-well plate and 96-well plate format. MagMA Microbiome Ultra Nucleic Acid Isolation Kit with proprietary bead tubes (ThermoFisher Scientific, Catalog no: A42358) was used with the automated extractor. All plates were prefilled manually with wash buffer (designated as Wash 1 or Wash 2 for each plate) along with 10 µL of RNase (1 mg/mL) per well, and two additional washes were filled with 1 mL of 80% Ethanol (designated as Wash 3 and Wash 4). Another plate was filled with elution buffer (100 µL per sample).

Briefly, the process is as follows: each sample was mixed in 800 µL of lysis buffer and 10 µL of Proteinase K. Homogenized samples were centrifuged for two minutes. Next, 400 µL of each lysed sample was transferred to a new plate, which was prefilled with 520 µL of binding bead mix. The plates were arranged in the following sequence for scanning and processing: Sample plate, Wash 1 plate, Wash 2 plate, Wash 3 plate, Wash 4 plate, Elution plate, and Tip Comb. The program was executed as follows: 300 s for DNA binding with the bead mix, followed by 60 s for Wash 1, 60 s for Wash 2, 40 s for Wash 3, 30 s for Wash 4, 120 s for drying beads, and finally, incubated at 75 °C for 150 s with 100 µL of elution buffer. The entire process took approximately 39 min. Upon completion of the run, the Elute plate was removed, samples were spun down at 300 rpm for 30 s and 100 µL of eluted DNA was transferred to a clean 1.5 mL microtube per sample.
