# Supplementary material for: A Comparison of Three Automated Nucleic Acid Extraction Systems for Human Stool Samples
Source: Microorganisms. 2024 Nov 25;12(12):2417. doi: 10.3390/microorganisms12122417 (PMC11678849; doi:10.3390/microorganisms12122417)
Supplement: Supplementary file 1 [file microorganisms-12-02417-s001.zip › microorganisms-3268932-supplementary.pdf]

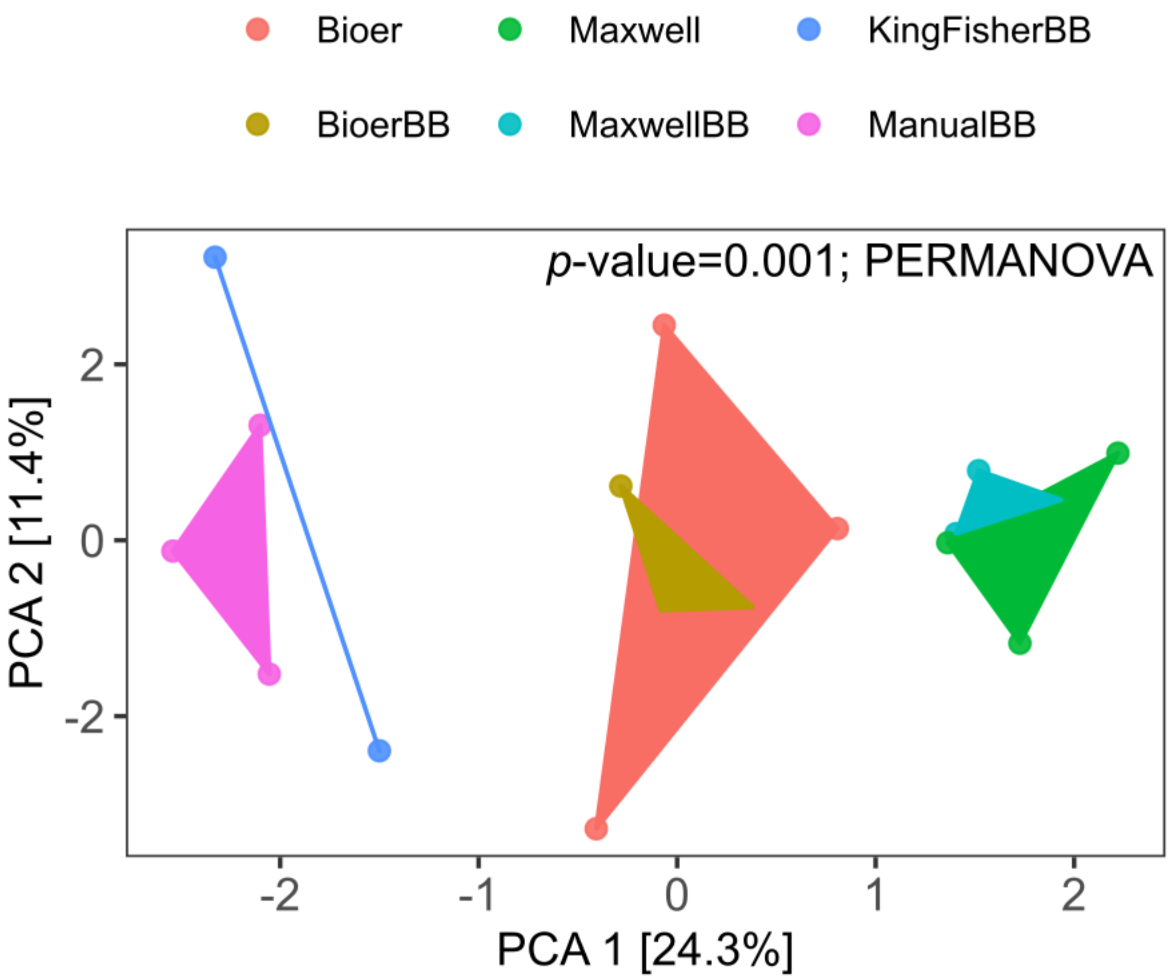


Figure S1. Principal component analysis (PCA) plot based on Aitchison distance of the mock community using different methods. The mock community consists of technical triplicates for each extraction method, except for KingFisher with duplicate samples. Sample points are adjoined by color-matched eclipses. The variance for each axis is shown in brackets.

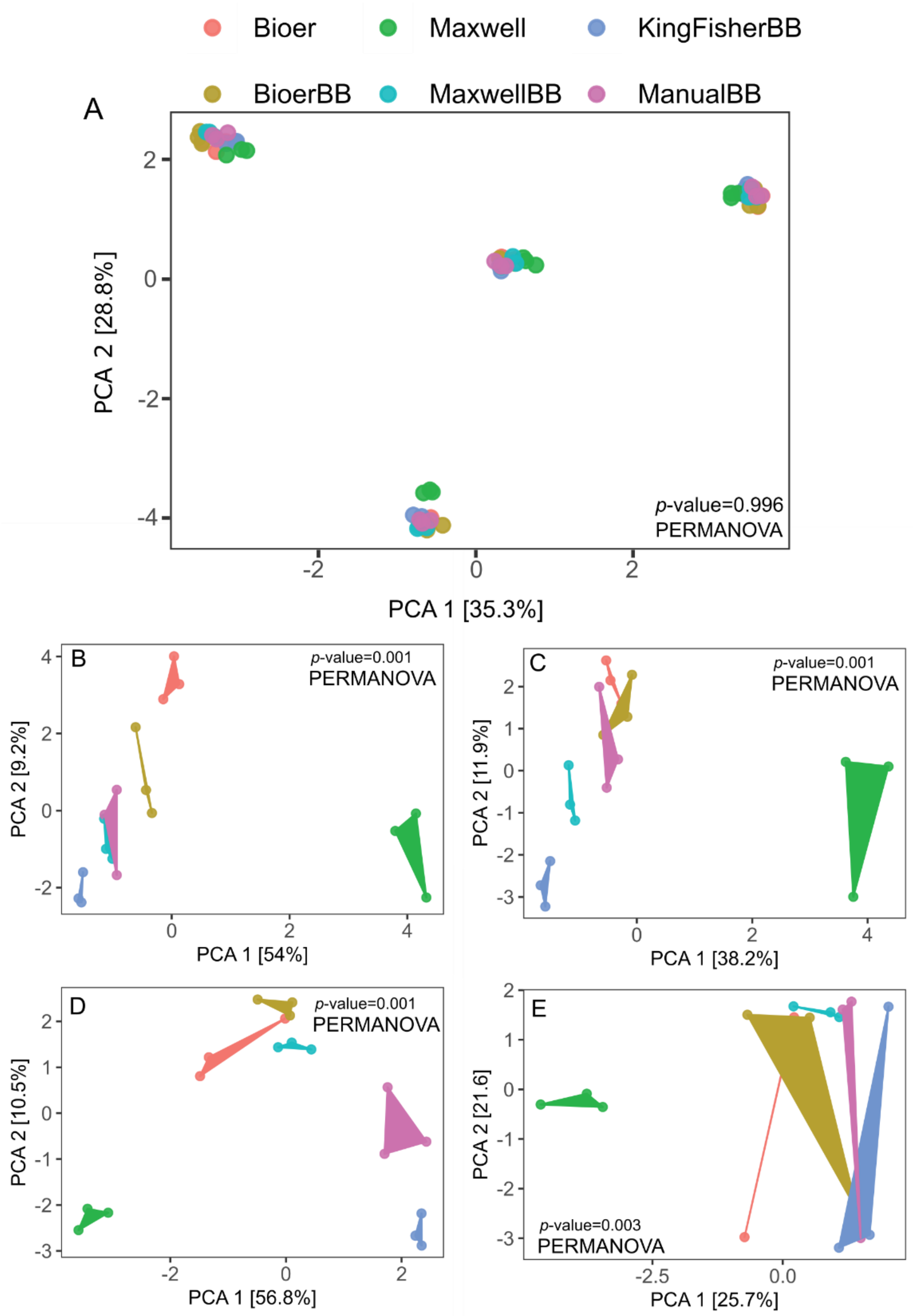


Figure S2. Principal component analysis (PCA) plots based on Aitchison distance among the fecal samples of different methods. A) PCA depicting the four biological samples (n=12 per method). B-E) PCA plots representing HC1, HC2, HC3, and HC4, respectively. The Aitchison distances of each biological sample with technical replicates (n=3 per method). The *p*-adjusted values for all pairwise PERMANOVA comparisons are not statistically significant (*p*-adjusted >0.05) and are not shown. The *p*-value for the overall PERMANOVA test for each PCA is indicated in the plot. The variance for each axis is shown in brackets.
